# History Leaves Its Mark on Soil Bacterial Diversity

**DOI:** 10.1128/mBio.00784-16

**Published:** 2016-06-07

**Authors:** Jennifer B. H. Martiny

**Affiliations:** Department of Ecology and Evolutionary Biology, University of California, Irvine, Irvine, California, USA

## Abstract

Dispersal is closely tied to the origin and maintenance of microbial diversity. With its focus on a narrow group of soil bacteria, recent work by Andam and colleagues on *Streptomyces* has provided perhaps the strongest support so far that some bacterial diversity in soils can be attributed to regional endemism (C. P. Andam et al., mBio 7:e02200-15, 2016, http://dx.doi.org/10.1128/mBio.02200-15). This means that dispersal is limited enough to allow for evolutionary diversification. Further analyses suggest that signatures of climate conditions more than 10,000 years ago can be detected in contemporary populations of this genus. These legacies have implications for how future climate change might alter soil microbial diversity.

## COMMENTARY

A long-held tenet in microbiology is that “everything is everywhere” (1). Indeed, many microbial taxa, often defined by ≥97% similarity of rRNA sequences, are widely distributed across the planet. The same taxa can be found on different continents, in separate ocean basins, and at both poles. These examples highlight the large geographic ranges of many microbial taxa and have been used as support for the idea that microbes are not dispersal limited. One implication of unlimited dispersal, or the complete mixing of microbial populations, is that any influence of past conditions on microbial diversity would be immediately erased by contemporary conditions.

Dispersal limitation is key to understanding the origin and maintenance of biodiversity—microbial and otherwise. In general, restricted dispersal between populations contributes to evolutionary diversification. If gene flow is restricted, then chance events within populations can contribute to population divergence. These events include unique mutations, the random order in which mutations arise, and genetic drift (2). Even the outcome of local adaptation to similar environments might differ between populations, because selection acts on these chance events. In this way, regional endemism arises, and overall diversity is greater than that of a globally mixed population.

The recent paper by Andam et al. in *mBio* (3) provides perhaps the strongest support so far that some of the enormous genetic diversity of soil bacteria can be attributed to regional endemism. Like many other studies on microbial biogeography (2), Andam and colleagues documented a distance-decay curve which showed that the similarity of bacterial composition between a pair of samples declines with the geographic distance between the samples (3). They further showed that this relationship was not well explained by the environmental conditions measured at the sampling locations. However, unlike most other studies, Andam et al. focused on a narrow group of bacteria, the genus *Streptomyces.* To do this, they selectively isolated strains from soil samples and characterized sample diversity on a relatively fine scale by sequencing the *rpoB* gene.

The choice to focus on a fine genetic scale was a key feature of the study of Andam et al., as it made it much more likely to detect genetic divergence among *Streptomyces* populations. Most microbial biogeographic studies have characterized microbial diversity by sequencing rRNA genes. The sequence diversity is then typically classified into taxa by using a 97% sequence similarity cutoff. However, based on rough estimates of 16S rRNA evolution (4), bacterial strains that differ by 3% in this gene diverged roughly 150 million years ago, around the time of the break up of the Gondwana supercontinent. To put this into perspective, contemporary birds evolved around 100 million years ago. Thus, if we used a similar genetic resolution for vertebrates, we would conclude that there is no regional endemism, because birds are present on all continents. (As an aside, even if bacteria do not display endemism on this genetic scale, dispersal limitation can still contribute to the maintenance of microbial diversity at the community level through ecological drift [5, 6]. However, this ecological effect of dispersal limitation is distinct from the origination of new, endemic diversity.)

The *Streptomyces* distance-decay curve, characterized on a fine genetic scale, is therefore consistent with regional endemism. On its own, this pattern is intriguing but ambiguous; it might be driven by unmeasured environmental variables. However, the authors’ conclusion was greatly strengthened by additional analyses. In particular, the authors found support for tropical niche conservatism within the genus. This hypothesis proposes that most diversity originated in the tropics, and later colonized nontropical regions, and glacial-interglacial cycles further reinforced these patterns. Consistent with this, *Streptomyces* diversity increases with decreasing latitude, and haplotype distributions appear to reflect recent glacial retreat (10,000 to 30,000 years ago). Thus, past climates seem to have left their signature on current *Streptomyces* diversity patterns. Moreover, mixing of *Streptomyces* populations within the United States has not erased these patterns, even after tens of thousands of years.

The Andam et al. study is also notable because it focused on soil bacteria. Earlier examples of microbial endemism primarily involved microbes that thrive in “island” habitats, such as animal hosts or terrestrial hot springs. In these habitats, it is not too difficult to imagine how microbial dispersal is limited. Successful migration of an individual between its habitats is highly unlikely; a cell must traverse a matrix of inhospitable habitats and, against the odds, encounter a relatively uncommon habitat. In contrast, barriers to dispersal in surface soils, which are common and contiguous, are harder to envision. However, the *Streptomyces* research suggests that when it comes to the dispersal of soil bacteria, our imagination may also be limited.

Finally, it is interesting to speculate what this study means for predicting changes in soil bacterial communities in the future. Terrestrial ecosystem models assume that microbial communities respond instantaneously to environmental change. However, the Andam et al. study results imply that some aspects of soil bacterial diversity may be due to legacies of historical conditions, even those of thousands of years ago. This would suggest that disturbances in the recent past—such as land use history within the last century—might leave even stronger signatures on contemporary soil diversity (5). Consideration of these legacies might therefore improve predictions of how microbial communities will respond to future environmental changes. Of course, predicting changes to microbial composition is just a first step; whether these changes will alter soil function is another open question.
